# Sleep deprivation and its effects on communication during individual and collaborative tasks

**DOI:** 10.1038/s41598-019-39271-6

**Published:** 2019-02-28

**Authors:** Benjamin C. Holding, Tina Sundelin, Mats Lekander, John Axelsson

**Affiliations:** 1 Department of Clinical Neuroscience, Karolinska Institutet, Stockholm, Sweden; 2 Department of Psychology, New York University, New York, USA; 3 Stress Research Institute, Stockholm University, Stockholm, Sweden

## Abstract

Sleep loss has been shown to cause impairments in a number of aspects central for successful communication, ranging from poorer linguistic comprehension to alterations in speech prosody. However, the effect of sleep loss on actual communication is unknown. This study investigated how a night of sleep deprivation affected performance during multiple tasks designed to test verbal communication. Healthy participants (N = 183) spent 8–9 hours per night in bed for three nights and were then randomised to either one night of total sleep deprivation or a fourth night with 8–9 hours in bed. The following day, participants completed two tasks together with another participant: a model-building task and a word-description task. Differences in performance of these tasks were assessed alongside speaking duration, speaking volume, and speaking volume consistency. Additionally, participants individually completed a verbal fluency assessment. Performance on the model-building task was worse if the model-builder was sleep deprived, whereas sleep deprivation in the instruction-giver predicted an improvement. Word-description, verbal fluency, speech duration, speaking volume, and speaking volume consistency were not affected. The results suggest that sleep deprivation leads to changes in communicative performance during instructive tasks, while simpler word-description tasks appear resilient.

## Introduction

Effective communication relies on diverse cognitive abilities, such as attention^1^, perspective taking^2^, and language ability^3^, and impairment of any of these abilities can decrease one’s communicative capacity^4,5^. This can in turn lead to decreased employee productivity^6^ or even more serious consequences, such as mistakes during medical procedures^7^. Since sleep loss leads to wide-ranging impairments in cognition^8^ and socio-emotional skills^9^, it is likely that communicative ability is also affected. However, the effect of sleep deprivation on communication in face-to-face social situations has yet to be investigated.

Sleep loss has been shown to lead to poorer performance on verbal fluency tasks^10,11^, which require individuals to spontaneously produce relevant words following a prompt, representing both executive functioning and linguistic ability^12^. However, the evidence is inconsistent and others have not found this impairment in verbal fluency^13^, or indeed found an improvement following sleep loss^14^. The reason for these inconsistencies may be related to the smaller samples (N range = 9–24) used in the studies showing significant effects. Therefore, the robustness and extent of this effect remains unclear.

Sleep deprivation may also decrease auditory and linguistic understanding. Sleep-deprived individuals have been shown to have worse understanding of grammatical structure^15^ and poorer language comprehension in long tasks^16^. Simulated night work has similarly been found to impair auditory attention and comprehension^17^, and there is evidence of poorer perception of speech in noisy environments following sleep deprivation, potentially relating to impairments in sensory gating^18^. This suggests that sleep loss impairs the ability to understand what others are saying, at least in longer tasks and when speech is less distinct.

The characteristics of speech may also be affected by sleep deprivation; people have been reported to slur^19^ and pause more^20^, as well as speak more monotonously^10^, more slowly^19,20^, and with less energy/intensity^21^ when sleep deprived. However, these effects have tended to be small and the studies run in solitary lab conditions. Therefore, the impact these changes have on person-to-person communication is still unknown.

Overall, there is a considerable overlap between abilities impaired by sleep loss and those required for successful communication. However, there is a dearth of relevant research in actual interpersonal situations. Further complicating this area is the difficulty of quantifying communication success. One study reported that following a night-shift, doctors showed a small number of specific conversational changes, such as being less likely to ask for clarification when conducting interpersonal interviews^22^. However, conclusions about the effect of sleep loss are confounded by the effects of longer working hours and increased work demand. Experimental research is necessary to account for such confounds.

In this study, we primarily aimed to determine whether sleep deprivation affects the ability to communicate efficiently during a dyadic interaction. We used two distinct collaborative tasks to capture broad changes in the ability to provide and understand verbal information. Since these tasks are assumed to rely on verbal communication, we hypothesised that the impact of sleep deprivation would lead to a decrease in task performance. During these tasks we also explored changes in the volume and duration of individuals’ speech. Using the same large sample, we also aimed to replicate the effects of sleep loss on individual verbal fluency seen in previous studies.

## Results

Descriptive statistics can be found in Table 1 for individual measurements and in Table 2 for dyadic task performance. For the first three nights, actigraphy showed similar sleep duration on average between participants who were later placed in the control condition (mean = 7:49 hours, SD = 51 minutes) compared to those who were placed in the sleep-deprivation condition (mean = 7:52 hours, SD = 55 minutes). On the night prior to testing, participants in the control condition were in bed for 8:18 hours (SD = 44 minutes) and slept for 7:51 hours (SD = 54 minutes).

**Table 1:** Mean and standard deviation (in brackets) for individual measures.

Measure	Outcome	Control	Sleep deprived
Verbal Fluency	FAS task score	37.76 (10.96)	40.97 (11.66)
	FAS task error	2.69 (3.29)	2.09 (2.27)
	Verb task score	18.96 (5.84)	19.07 (6.65)
	Verb task error	1.35 (1.05)	1.24 (1.07)
Speech	Speaking duration (seconds)	649.69 (127.37)	616.13 (116.40)
	Speech volume (z-score)	0.01 (1.05)	−0.01 (0.95)
	Speech volume consistency (z-score)	−0.04 (0.99)	0.05 (1.01)

Note. For verbal fluency: control condition N = 89, sleep deprivation condition N = 91. For speech: control condition N = 88, sleep deprivation condition N = 85.

**Table 2:** Mean and standard deviation (in brackets) for group task performance.

Task	Outcome	Roll/condition combinations				
**Model-building**		Describer:	Control	Control	Sleep deprived	Sleep deprived
		Builder:	Control	Sleep deprived	Control	Sleep deprived
		N_dyad_:	30	15	16	28
	Score		6.50 (2.75)	4.20 (3.10)	7.13 (2.96)	6.14 (2.64)
	Time taken (seconds)		519.63 (90.09)	524.07 (110.48)	506.63 (100.31)	528.04 (90.11)
	Efficiency		0.12 (0.92)	−0.53 (1.06)	0.38 (1.07)	0.00 (0.95)
**Word-description**		Speaker:	Control	Control	Sleep deprived	Sleep deprived
		Guesser:	Control	Sleep deprived	Control	Sleep deprived
		N_round_:	180	93	93	174
	Score (per round)		1.88 (1.71)	1.73 (1.15)	1.84 (1.72)	1.82 (1.31)

Note. The mixed condition combinations in the word-description task are related to specific rounds (due to alternation of roles) rather than separate dyads. For model-building: sleep-deprived describer N = 44, control describer N = 45, sleep-deprived builder N = 43, control builder N = 46. For word-description: sleep deprivation condition N = 89, control condition N = 91.

### Verbal fluency

Neither of the verbal fluency tasks revealed any differences in words produced or number of errors between the sleep-deprived and control groups (see Table 3).

**Table 3:** Bayesian t-test of differences between sleep conditions for verbal fluency.

Outcome	Control sleep		Sleep deprivation		Difference			
	Mu	Sigma	Mu	Sigma	Mu	HDI low	HDI High	Effect size
FAS task Score	37.71	10.59	40.81	11.45	3.10	−0.23	6.47	0.28
FAS task Errors	1.54	1.52	1.44	1.36	−0.10	−0.66	0.46	−0.07
Verb task Score	19.06	5.45	18.79	6.07	−0.26	−2.12	1.56	−0.05
Verb task Errors	1.31	0.89	1.18	0.94	−0.13	−0.42	0.17	−0.15

Note. Mu = posterior distribution mean; Sigma = posterior distribution residual standard deviation; HDI = 95% Highest Density Interval; Effect Size = (mu1 - mu2)/sqrt((sigma1^2^ + sigma2^2^)/2)^38^. FAS score prior: population mean = 42, sigma = 21. Verb score prior: population mean = 20, sigma = 10. Non-informative (default) priors were used on the number of errors since we had no data to base this on.

### Model-building

Using overall score (number of bricks correctly placed) as the predicted variable, the data suggest that sleep deprivation lead to changes in performance for both the builder and describer roles. The median estimate shows that sleep deprivation in builders caused a decrease of 1.18 (13%) in task score. On the other hand, sleep deprivation in the describer was associated with an *increase* of 0.87 (10%) in task score. Sleep deprivation in either role did not predict time taken to complete the task; however, sleep deprivation in builders was found to decrease task efficiency, with fewer points per second. For describers, this effect was not distinguishable from zero. The full results for this task can be seen in Table 4.

**Table 4:** Bayesian regression estimating the effect of sleep deprivation on model-building performance.

Outcome	Describer sleep-deprivation effect				Builder sleep-deprivation effect			
	Mu	HDI low	HDI high	Sigma	Mu	HDI low	HDI high	Sigma
Score	**0.87**	**0.00**	**1.67**	**0.42**	**−1.18**	**−1.99**	**−0.33**	**0.42**
Time taken (seconds)	−4.63	−48.60	36.00	21.63	12.72	−27.44	54.19	21.14
Efficiency (z-score)	0.31	−0.08	0.69	0.20	**−0.41**	**−0.79**	**−0.05**	**0.19**

Note. Mu = posterior distribution mean; Sigma = posterior distribution residual standard deviation; HDI = 95% Highest Density Interval; Bold rows represent changes in performance that can be considered ‘significant’. Score model priors: mean effect = 0, sigma = 2.25, limited between −9 and 9. Time-taken priors: mean effect = 0, sigma = 150, limited between −600 and 600. Efficiency model priors: mean effect = 0, sigma = 0.5, limited between −2 and 2. A cumulative distribution was used for the score response outcome, while a Gaussian distribution was used for time and efficiency outcomes.

### Word-description

Sleep deprivation did not predict a change in score per round for either guesser or speaker performance (see Table 5).

**Table 5:** Bayesian multilevel regression estimating the effect of sleep deprivation on word-description performance.

Outcome	Speaker sleep-deprivation effect				Guesser sleep-deprivation effect			
	Mu	HDI low	HDI high	Sigma	Mu	HDI low	HDI high	Sigma
Score (per round)	0.00	−0.42	0.43	0.22	−0.12	−0.55	0.27	0.21

Note. Mu = posterior distribution mean; HDI = 95% Highest Density Interval; Sigma = posterior distribution residual standard deviation. Priors were set on the effect; since there was no maximum possible effect, sigma was set as a quarter of maximum observed round score (mean = 0, sigma = 1.5). The response distribution was set to cumulative since model fit comparisons revealed that this was the best fit for the data.

### Speaking duration, volume and volume consistency

No difference was found between the sleep-deprived and control groups in speaking duration, average speaking volume, or speaking volume consistency (see Table 6).

**Table 6:** Bayesian t-test of differences between sleep conditions for speech characteristics.

Outcome	Control sleep		Sleep deprivation		Difference			
	Mu	sigma	Mu	sigma	Mu	HDI low	HDI High	Effect size
Speaking duration (seconds)	649.15	123.65	617.48	113.69	−31.67	−68.43	5.42	−0.27
Speech volume (z-score)	−0.08	0.90	−0.10	0.82	−0.02	−0.31	0.26	−0.02
Speech volume consistency (z-score)	−0.05	0.98	0.03	1.00	0.09	−0.22	0.40	0.09

Note. Mu = posterior distribution mean; Sigma = posterior distribution residual standard deviation; HDI = 95% Highest Density Interval; Effect Size = (mu1 - mu2)/sqrt((sigma1^2^ + sigma2^2^)/2)^38^. Speaking duration prior: mean = 1050, sigma = 525. Non-informative (default) priors were used on speech volume and speech volume consistency, since no studies have been published that reported raw values.

## Discussion

In this study, we investigated the impact of a single night of total sleep deprivation on the ability to communicate. Using individual verbal fluency tasks, no noticeable difference was found between those who were sleep deprived and those who had slept sufficiently. This sets our results apart from some previous studies that have shown verbal fluency to be impaired following sleep deprivation^10,11^. Indeed, for the FAS task, the majority of the posterior distribution is greater than zero, suggesting that if anything, sleep deprivation is more likely to have a *beneficial* effect on verbal fluency. This corresponds with one previous study^14^, reporting an improvement in verbal fluency following sleep deprivation. Our study differs from most studies in that participants were only measured at one timepoint, while others have used repeated measures. Additionally, task duration varies between previous studies, which may explain the differences in result. However, the convergence of evidence between this study and the largest other study conducted^13^, suggests that the effect of sleep deprivation on verbal fluency is less robust than earlier findings indicated.

Performance on the model-building task was affected by sleep deprivation. If the builder was sleep deprived, overall performance of the dyad was lower. This follows previous research suggesting that verbal perception and linguistic comprehension are decreased after sleep loss^15–18^. We also found that task efficiency (the number of bricks correctly placed per second) was lower if the builder was sleep deprived. Since builder sleep deprivation did not predict time taken to complete the task, the change in efficiency for builders can be assumed to be driven by the change in information-flow rate rather than a change in time-on-task.

The improvement in model-building score if the describer was sleep deprived was unexpected. A speculative explanation is that since sleep deprivation may increase reactivity to psychosocial stressors^23^ (though evidence is inconsistent)^24^, and stress can facilitate cognitive processes such as allocentric spatial processing^25^, such an increase in stress levels could have been beneficial for performance in this role. Alternatively, aspects of speech prosody not measured could have made understanding the instructions easier. For example, though our data shows no change in total speaking duration after sleep deprivation, it is possible that participants spoke fewer words while at the same time speaking more slowly. This could conceivably boost intelligibility by information becoming more direct and easier to understand.

Sleep deprivation did not have a noticeable effect on performance on the word-description task, nor was there any evidence of either additive or interaction effects. Compared to the model-building task, the word-description task allows for full attention to be directed at the speaker, rather than being split between the speaker and the building bricks. This may explain the different outcomes, as sleep-deprived people have impaired attentional abilities^8^. Furthermore, the brevity of the word-description task may make it less susceptible to failures in attention. Participants could also see each other’s facial expressions, gaining non-verbal information which may help to counteract the effects of impaired verbal communication ability.

Sleep deprivation did not predict the overall amount of speech, speech volume, or speech volume consistency during the dyad interactions. This suggests that although previous laboratory observations have found altered speech after sleep loss^19,20^, speech contribution in actual interpersonal situations may not be impacted. However, the posterior distribution is generally too wide to exclude the possibility of small effects and there may be other aspects of prosody that are affected by sleep loss, such as intonation^10^.

There are a number of limitations to this study and potential directions for future research. The tasks were generally short in duration, and given that one of the strongest impairments following sleep loss is in sustained attention^8^, communicative tasks that require concentration over longer periods of time may be increasingly impacted. A further limitation is that the interactions were between two unacquainted individuals. Interactions with strangers have greater novelty and are potentially more physiologically arousing than with known individuals^26^, possibly overriding the effects of sleep loss. Additionally, while Sociometric Badges (or similar devices) hold promise as a research tool, the pre-processing of the audio and the proprietary algorithms that control the output mean that it was not possible to analyse the raw audio signal, which may have led to more comprehensive insights about changes in speech prosody after sleep deprivation. More broadly, it is difficult to quantify communication ability, since the definition of performance depends heavily on the situation. In this study, we have aimed to address this issue by investigating the effect on sleep deprivation on communication in different ways, specifically using the performance on three verbal tasks as a marker for communicative ability. Future research would benefit from more closely investigating the mechanisms behind the changes seen in the model-building task, as well as the generalisability of performance impairments to other collaborative situations.

Overall, we find that sleep-deprived participants were less efficient and less accurate when acting on instructions to build an abstract model. Conversely, when a sleep-deprived person gave instructions, performance was improved. These effects were found despite a lack of effect of sleep deprivation on word-description, verbal fluency, speech duration, or speech volume. Since many occupations rely on the ability to quickly understand and act on instructions, it is important for future studies to clarify the mechanisms behind these changes. An impairment in successfully following instructions could have important implications for a wide range of work environments such as on-call medical staff or air transport, where sleep loss is common.

## Method

### Participants

The sample consisted of 183 individuals (104 women; mean age = 25.36 years, SD = 6.49 years) after the exclusion of one participant due to being outside of our age range requirement (18–45 years) at the time of testing. Potential participants were recruited via posters/online, screened using an online questionnaire and excluded if found to have habitually poor sleep, health problems, or high alcohol/tobacco/caffeine usage. A complete list of exclusion criteria can be found in the supplementary materials. Due to participant dropout, two of the participants were short-notice replacements. Though the sleep of these two participants was not measured as in other participants, they both verbally reported sleeping sufficiently and were analysed as control (normal sleep) participants in the collaborative tasks (see Tables S1 and S2 for results with these two dyads removed). The final sample was 92 control and 91 sleep-deprived participants. The study was approved by the Stockholm Regional Ethical Review Board (no. 2014/1766-32), complied with relevant ethical standards, and obtained informed consent from all participants. Participants were compensated for taking part (1500SEK for sleep deprived participants, 800SEK for control participants).

### Measurements

#### Verbal fluency

Verbal fluency was assessed using Swedish variants of the Controlled Oral Word Association Test^27^, specifically the FAS Swedish Letter Task, and the Swedish Verb Task^28^. The FAS task required participants to say as many words as possible that begin with a specified letter (F, A, and S). Participants had 60 seconds for each letter. Proper nouns or the same words with different endings (e.g. “lamp”, “lampshade”) were not allowed. The verb task required participants to say as many different verbs as possible in 60 seconds. Verbs with different endings/tenses (e.g. “walk”, “walked”) or the same word used in different combinations (e.g. “go away”, “go home”) were not allowed. The outcome was the total amount of words produced, excluding words that broke the above rules. A greater total score represents better verbal fluency. We also investigated the total amount of errors (words that broke the above rules). All sessions were audio recorded, and subsequently scored by trained examiners, naïve to the condition of the participant.

#### Model-building

**Figure 1:**
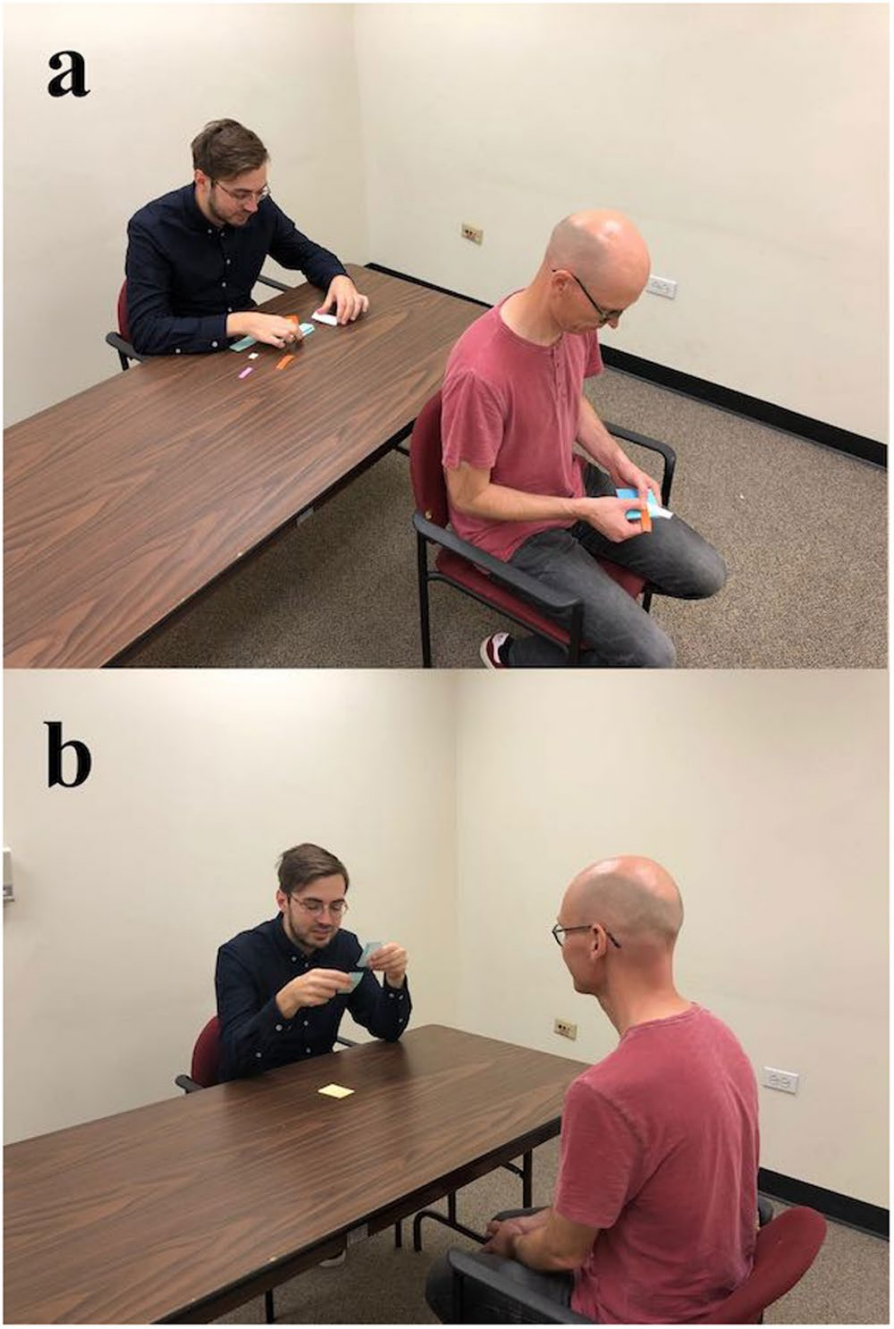
A representative illustration of the seating arrangement during the two collaborative tasks. (**a**) Model-building task setup with builder (left) and describer (right). (**b**) Word-description task setup with speaker (left) and guesser (right). Note that Sociometric Badges (not shown) were additionally worn during all collaborative tasks.

**Figure 2:**
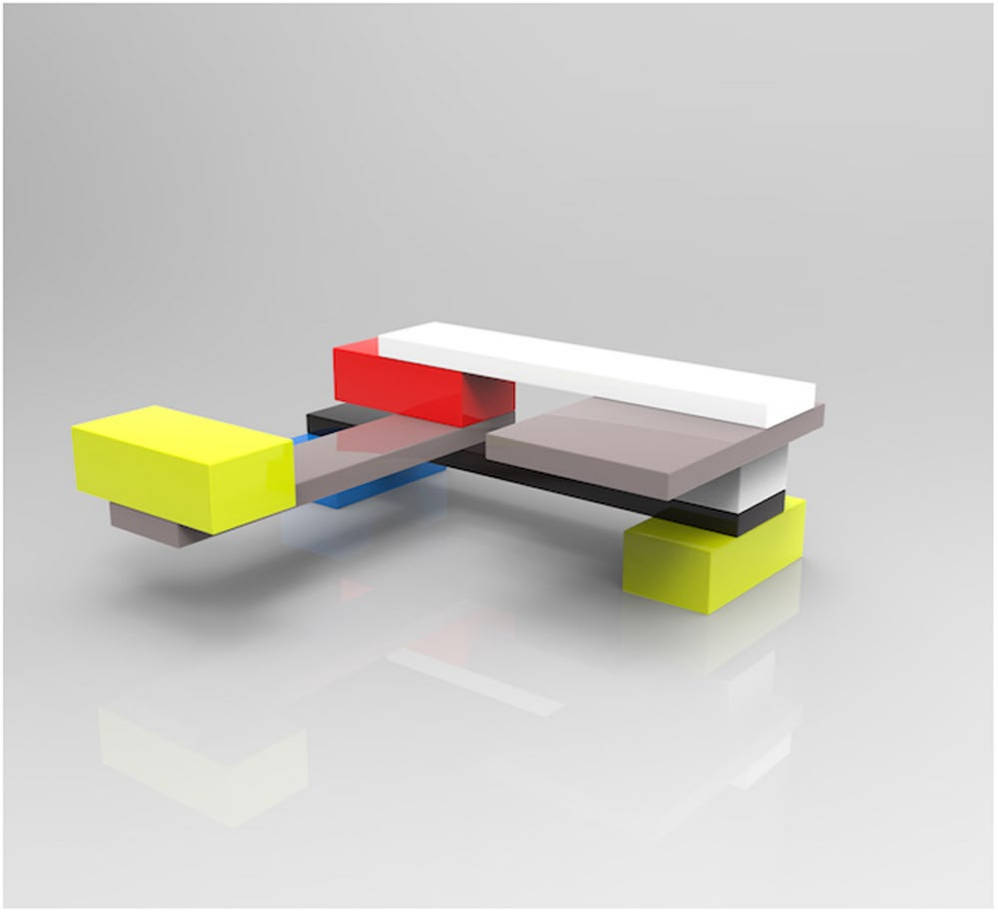
A graphical representation of the target model used in the model-building task.

A two-person model-building task was used to assess verbal communication^29,30^. In pairs, participants were randomly assigned to be either a ‘*describer’* or a ‘*builder’*. Participants sat at opposite ends of a small table and the describer was instructed to turn their chair around 180-degrees so that participants were not sitting face-to-face (see Fig. 1a). The describer was then given an abstractly shaped model built from plastic interlocking building bricks and asked to instruct their partner to build an identical model, using verbal instructions only. The builder could not see the model nor the face of the describer. The model was the same for each pair and consisted of 9 bricks of different colours (see Fig. 2). The builder was provided with 30 bricks of varying shapes and colours, and it was not possible to identify a brick with a single feature (e.g. ‘the red one’). Each pair had 10 minutes to solve the task and they were allowed to speak freely. The task provides flexibility for the participants to solve the problem in their own way, while allowing quantification of success in terms of the number of pieces correctly placed. The main outcomes for this task were the pair’s score (number of bricks correctly placed), the time taken to complete the task (in seconds, max 600), and a standardised z-score of efficiency (score/time taken to complete task).

#### Word-description

The ability to describe words was tested using the game Taboo (Hasbro, Pawtucket, Rhode Island). During this game, participants attempted to describe as many target words as possible to their partner in 60 seconds, without saying the target word. Each word-prompt card contained the target word, plus an additional five related words that were prohibited. Participants sat face-to-face across a table, while keeping the word cards hidden from each other (see Fig. 1b). Pairs took turns so that each participant acted three times as the ‘*speaker’* and three times as the ‘*guesser’*. There were 40 different word cards and the order was randomised for each pair. The amount of correct words per pair was later scored using a video recording of the task. For every word guessed correctly, both participants in the pair later received 5 SEK (approximately $0.60). The outcome variable was the total score for each of the six rounds.

#### Speaking duration, volume and volume consistency

Each participant wore a Sociometric Badge (Sociometric Solutions Inc., Boston, MA, USA) while interacting with their partner. These devices provide a non-intrusive and objective method to measure speech patterns of the wearer during face-to-face interaction, but rather than recording conversations the devices automatically analyse the physical characteristics of speech. The influence of extraneous noise is reduced by excluding frequencies outside of the human speech range, and the active speaker is defined as the badge with the highest speech amplitude at that moment. Further technical details are available in previous validation studies of Sociometric Badges^31–34^. In this study, speech patterns were assessed over the entire dyadic interaction session (approximately 40 minutes, varying slightly depending on the time it took each pair to finish the tasks). The outcome values for each participant are the total speaking duration (in seconds, including overlapping speech), mean volume, and mean volume consistency. Since the unit of measurement for volume is not on a standard scale, we created standardised z-scores for both mean volume and mean volume consistency as previously recommended^31^.

### Procedure

Participants were asked to be in bed for 8–9 hours, and attempt to sleep for 8 hours for three nights prior to testing. They were instructed to go to sleep (i.e. turn off the lights) at 23:00 ± 60 min and get up at 07:00 ± 60 min. To check for differences in prior sleep between groups, we assessed the sleep of participants using sleep diaries (Karolinska Sleep Diary)^35^ and actigraphy (GeneActiv Sleep, Activinsights Ltd, Kimbolton). Actigraphy data was scored using the Actant activity analysis toolbox (https://github.com/btlindert/actant-1) for MATLAB (The MathWorks Inc., Natick, MA, USA). For 11 participants, due to device failure, an alternative actigraph was used (MotionWatch 8 scored with MotionWare software, CamNtech, Cambridge, UK). During this period, participants were asked not to nap, abstain from alcohol, and not consume caffeine past the morning of the penultimate day (day before testing).

On the penultimate day, participants were informed via telephone whether they would be required to come to the lab at 22:00 that night and stay awake, or sleep one more night at home and arrive at 10:00 the following day. The condition of each participant was randomised (while keeping an equal number of participants in each condition). Participants in the sleep-deprivation condition were kept in a light-controlled room with a research assistant, and could freely choose their activities throughout the night (e.g. study, watch a film). Low-sugar snacks were available during the night, and breakfast was provided in the morning. A morning stroll of 15–20 minutes was taken to imitate the journey from the home to the lab.

All tasks were led by experimenters blind to the condition of the participants. Verbal fluency was assessed at approximately 10:30 and the dyadic tasks begun at approximately 13:00. Participants first encountered their partner at the beginning of the dyadic segment, when they were seated opposite each other at a small table, and instructed to wear the Sociometric Badge. They continued to wear this device for the entire dyadic segment.

The first 15 minutes were spent on a group decision task (to be published separately). Subsequently, participants completed the model-building task, and lastly the word-description task. Participants did not receive feedback about their performance on the tasks. The pairs could consist of any combination of the sleep conditions (i.e. sleep deprived-sleep deprived, sleep deprived-control, control-control). All tasks were conducted with the experimenter outside of the room. Timing began as soon as the experimenter had finished giving instructions and exited the room.

### Data analysis

The data were analysed using Bayesian statistics in R^36^ (DOI for data and code: 10.5281/zenodo.1239717). A Bayesian approach encourages probability statements rather than *p*-values, and allows for acceptance of the null hypotheses, unlike traditional statistics, which can only inform rejection decisions^37^. For individual measurements (i.e. verbal fluency tasks and speech characteristics), the BEST^38^ package was used for two-group comparisons (a ‘Bayesian t-test’), providing estimates for the differences between conditions. For dyadic tasks (i.e. model-building and word-description), the brms^39^ package was used to create separate linear models (a ‘Bayesian regression’). For the model-building tasks, the sleep condition of builders and describers were added as two dummy predictors (well-rested = 0, sleep deprived = 1) in models for each performance outcome (score, time taken, efficiency). For the word-description task, speaker and guesser sleep condition dummy variables were predictors in a model predicting the score of each round. To account for the non-independence of residual variance within each dyad, random intercepts were added for each pair. More complex random-effects structures were not possible without convergence problems biasing the posterior sample.

As is the norm for Bayesian statistics, priors were set on certain model parameters. For group comparisons, the prior consisted of overall mean based on known existing data^28^ and a standard deviation of half of this mean. For the regression models, a prior distribution (representing expectations under the null hypothesis) was set on the effect. This was always zero, with a standard deviation of a quarter of the maximum possible effect. All other parameters used the default non-informative priors of the R package^38,39^. Exact prior specifications can be found in the notes of Tables 3–6. Model fit comparisons using the Watanabe-Akaike Information Criterion and Leave-One-Out were used for determining the best-fitting probability distribution for each model, and whether to include a fixed effect interaction between predictors. Since model fit did not improve after fitting interaction effects, and our hypotheses were focused on the main effects, we chose not to include the interaction effects in the final models.

Decision rules regarding the acceptance or rejection of hypotheses were based on the posterior distribution’s 95% highest density interval (HDI)^40^. Acceptance of the alternative hypothesis (a ‘significant’ result) was made if the 95% HDI did not overlap zero, while for values that lay outside the 95% HDI the null hypothesis was accepted^41^. For values in between, more data are needed to either accept or reject the hypothesis.

There was a small amount of data loss during the study. One control participant did not complete the verbal fluency tasks, due to finding it too stressful. Two pairs (2x both sleep deprived) were removed from the model-building task analysis, due to a logistical error allowing the pairs to take longer than 10 minutes. A technical problem with the recording equipment meant that it was not possible to score the word-description task for one pair (both sleep deprived). A further pair (both control) was removed from both the model-building and word-description task due to one participant being outside of the 18–45 year age range at testing. The sociometric data of this participant were also excluded, along with that of 8 other participants (2 control, 6 sleep deprived) that were lost due to technical problems with the Sociometric Badges.

## Supplementary information

Supplementary materials

## References

[1] Sperber D, Wilson D (1987). Précis of relevance: Communication and cognition. Behav. Brain Sci..

[2] Krauss RM, Fussell SR (1991). Perspective-taking in communication: Representations of others’ knowledge in reference. Soc. Cogn..

[3] Littlemore J, Low G (2006). Metaphoric competence, second language learning, and communicative language ability. Appl. Linguist..

[4] Docherty NM (1996). Working memory, attention, and communication disturbances in schizophrenia. J. Abnorm. Psychol..

[5] Happé FGE (1993). Communicative competence and theory of mind in autism: A test of relevance theory. Cognition.

[6] Henderson LS (2008). The impact of project managers’ communication competencies: Validation and extension of a research model for virtuality, satisfaction, and productivity on project teams. Proj. Manag. J..

[7] Greenberg CC (2007). Patterns of communication breakdowns resulting in injury to surgical patients. J. Am. Coll. Surg..

[8] Lim J, Dinges DF (2010). A meta-analysis of the impact of short-term sleep deprivation on cognitive variables. Psychol. Bull..

[9] Beattie L, Kyle SD, Espie CA, Biello SM (2015). Social interactions, emotion and sleep: A systematic review and research agenda. Sleep Med. Rev..

[10] Harrison Y, Horne JA (1997). Sleep deprivation affects speech. Sleep.

[11] Horne JA (1988). Sleep loss and ‘divergent’ thinking ability. Sleep.

[12] Aita, S. L. *et al*. Executive, language, or both? An examination of the construct validity of verbal fluency measures. *Appl. Neuropsychol. Adult* 1–11, 10.1080/23279095.2018.1439830 (2018). 10.1080/23279095.2018.1439830 29513079

[13] Binks PG, Waters WF, Hurry M (1999). Short-term total sleep deprivations does not selectively impair higher cortical functioning. Sleep.

[14] Tucker AM, Whitney P, Belenky G, Hinson JM, Van Dongen HPA (2010). Effects of sleep deprivation on dissociated components of executive functioning. Sleep.

[15] Kim DJ (2001). The effect of total sleep deprivation on cognitive functions in normal adult male subjects. Int. J. Neurosci..

[16] Pilcher JJ (2007). Language performance under sustained work and sleep deprivation conditions. Aviat. Sp. Environ. Med..

[17] Pilcher JJ, Jennings KS, Phillips GE, McCubbin JA (2016). Auditory attention and comprehension during a simulated night shift: Effects of task characteristics. Hum. Factors.

[18] Fostick L, Babkoff H, Zukerman G (2014). Effect of 24 hours of sleep deprivation on auditory and linguistic perception. J. Speech, Lang. Hear. Res..

[19] Honig, F. *et al*. Are men more sleepy than women or does it only look like - Automatic analysis of sleepy speech. In *2014 IEEE International Conference on Acoustics, Speech and Signal Processing (ICASSP)* 995–999, 10.1109/ICASSP.2014.6853746 (IEEE, 2014).

[20] Vogel AP, Fletcher J, Maruff P (2010). Acoustic analysis of the effects of sustained wakefulness on speech. J. Acoust. Soc. Am..

[21] McGlinchey EL (2011). The effect of sleep deprivation on vocal expression of emotion in adolescents and adults. Sleep.

[22] Liu CC, Wissow L (2011). How post-call resident doctors perform, feel and are perceived in out-patient clinics. Med. Educ..

[23] Minkel J (2014). Sleep deprivation potentiates HPA axis stress reactivity in healthy adults. Heal. Psychol..

[24] Schwarz J (2018). Does sleep deprivation increase the vulnerability to acute psychosocial stress in young and older adults?. Psychoneuroendocrinology.

[25] Cao Z, Wang Y, Zhang L (2017). Real-time acute stress facilitates allocentric spatial processing in a virtual fire disaster. Sci. Rep..

[26] Spitzer SB, Llabre MM, Ironson GH, Gellman MD, Schneiderman N (1992). The influence of social situations on ambulatory blood pressure. Psychosom. Med..

[27] Benton, A. L. & Hamsher, K. *Multilingual Aphasia Examination (MAE)*. (Aja Associates, 1978).

[28] Tallberg IM, Ivachova E, Jones Tinghag K, Östberg P (2008). Swedish norms for word fluency tests: FAS, animals and verbs. Scand. J. Psychol..

[29] Krych-Appelbaum M (2007). I think I know what you mean: The role of theory of mind in collaborative communication. Interact. Stud..

[30] Clark HH, Krych MA (2004). Speaking while monitoring addressees for understanding. J. Mem. Lang..

[31] Kayhan VO (2018). How honest are the signals? A protocol for validating wearable sensors. Behav. Res. Methods.

[32] Chaffin D (2017). The promise and perils of wearable sensors in organizational research. Organ. Res. Methods.

[33] Onnela J-P, Waber BN, Pentland A, Schnorf S, Lazer D (2014). Using sociometers to quantify social interaction patterns. Sci. Rep..

[34] Yu D (2016). Intelligent Emergency Department: Validation of Sociometers to Study Workload. J. Med. Syst..

[35] Åkerstedt T, Hume K, Minors D, Waterhouse J (1994). The subjective meaning of good sleep, an intraindividual approach using the Karolinska Sleep Diary. Percept. Mot. Skills.

[36] R Core Team. R: A language and environment for statistical computing (2016).

[37] Wagenmakers E, Morey RD, Lee MD (2016). Bayesian benefits for the pragmatic researcher. Curr. Dir. Psychol. Sci..

[38] Kruschke, J. K. & Meredith, M. BEST: Bayesian estimation supersedes the t test. *R package version 0.5.0*. at, https://cran.r-project.org/web/packages/BEST/vignettes/BEST.pdf (2018).

[39] Bürkner P-C (2017). brms: An R Package for Bayesian Multilevel Models Using Stan. J. Stat. Softw..

[40] van der Linden S, Chryst B (2017). No need for Bayes Factors: A fully Bayesian evidence synthesis. Front. Appl. Math. Stat..

[41] Kruschke JK (2013). Bayesian estimation supersedes the t test. J. Exp. Psychol. Gen..

