# Supplementary material for: Sleep deprivation and its effects on communication during individual and collaborative tasks
Source: Sci Rep. 2019 Feb 28;9:3131. doi: 10.1038/s41598-019-39271-6 (PMC6395705; doi:10.1038/s41598-019-39271-6)
Supplement: Supplementary file 1 — Supplementary materials [file 41598_2019_39271_MOESM1_ESM.pdf]

Suppmentary information and tables

Study title:

Sleep deprivation and its effects on communication during individual and collaborative tasks

Authors:

Benjamin C. Holding[1]

Tina Sundelin[1, 2]

Mats Lekander[1,3]

John Axelsson[1,3]

Affiliations:

[1]Department of Clinical Neuroscience, Karolinska Institutet, Sweden

[2]Department of Psychology, New York University, USA

[3]Stress Research Institute, Stockholm University, Sweden

Exclusion criteria from initial screening

<u>BACKGROUND</u>
Age
Exclude: Under 18 or over 45

BMI [based on height and weight]
Follow-up: 27+ use Epworth Sleepiness Scale (Johns, 1991) to assess excessive daytime sleepiness and exclude anyone scored 9 or more. If BMI is 30+ also use STOP-Bang (Chung et al, 2016) to assess sleep apnea and exclude those scoring 3 or more.

Studies
Exclude: Having studied psychology for a term (6 months) or more

Swedish language skills (alternatives: beginner, good, very good, fluent)
Exclude: Response of 'beginner' or 'good'.
Follow-up: 'very good' [contact and assess]

<u>SLEEP</u>
How do you generally judge your sleep quality? (alternatives: very good, quite good, neither good or bad, quite bad, very bad)
Exclude: Response of 'quite bad' or 'very bad'.

Have you had any of the following problems during the last 6 months? (alternatives: never or seldom, up to three times per month, one-three times per week, four times per week or more):

- *Difficulties falling asleep*
- *Difficulties waking up*
- *Repeated awakenings with difficulties to fall back to sleep*
- *Feeling exhausted when waking up*
- *Disturbed sleep*
- *Unintended sleep episodes (nodding off) during work or freetime*
- *Light and shallow sleep*
- *Respiratory arrest or difficulty breathing during sleep (according to yourself or others)?*
- *Feelings of restlessness in your legs when you are in bed or sleeping*

Exclude: 'one-three times per week' or 'four times per week or more'

- *Too little sleep (less than 6 hours)*

Exclude: 'four times per week or more'

How much sleep do you think that you need per day?
Exclude: below 7 hours or above 9 hours

Do you use any medicine or supplement to sleep better? (alternatives: Yes regularly, yes periodically, no)
Exclude: 'yes regularly' or 'yes periodically'

HEALTH

Have you been in contact with a doctor over any of the following (alternatives: no, yes currently, yes during the last year, yes more than a year ago):

- *Pain in the chest or back*
- *Disturbed sleep*
- *Depression*
- *Other psychiatric problem (e.g. anxiety, worry, sadness, etc.)*
- *Heart attack and / or high blood pressure*
- *Diabetes*
- *Other disease: [specify]*

Exclude: 'yes currently' or 'yes during the last year'

Do you take any medicine regularly?
Follow-up: If yes, research assistant investigates further. Contraceptive pill, weak pain medication is okay. Decision on a case-by-case basis.

What is your tobacco usage? (alternatives: do not smoke or use snus, previously smoker/snus-user (stopped more than 3 months ago), previously smoker/snus-user (stopped less than 3 months ago), current smoker or snus-user, smoke/use snus sometimes
Exclude: 'previously smoker/snus-user (stopped for less than 3 months ago)' or 'current smoker/snus-user'
Follow-up: 'Smoke/use snus sometimes' [check that the participant can abstain for more than a day].

How often do you drink alcohol? (alternatives: never, once a month or less, 2-4 times a month, 2-3 times a week, 4 times a week or more)
Follow-up: '2-3 times a week' or '4 times a week or more' [check what sort of strength the alcohol is. If weak beer (< 3.5%) okay, if strong spirit exclude]

How much coffee do you drink per day? (alternatives: none, 1-2 cups, 3-4 cups, 5-6 cups, 7 or more cups)
Exclude: '7 or more cups'
Follow-up: '3-4 cups', or '5-6 cups' [check whether they can abstain from caffeine without experiencing withdrawal-effects]

How often do you use drugs other than coffee, alcohol, or tobacco? (alternatives: never, once a month or less, 2-4 times per month, 2-3 times per week, 4 times per week or more)
Exclude: 2-4 times per month, 2-3 times per week, 4 times per week or more

OTHER QUESTIONS

Have you worked a night-shift during the last month? (alternatives: yes, no)
Follow-up: yes [must be more than 3 weeks before the testing day]

How well do the following statements characterise you? (alternatives: 0 – characterises very well, 0 - characterises quite well, 1 - characterises quite poorly, 2- doesn’t characterise me at all) [summed score for the following items]

- It is easy for me to ”read between the lines” when someone talks to me
- I can tell if a person listening to me is getting bored
- When I read a story it is difficult for me to understand the intentions of the characters*
- I find it easy to work out what someone is thinking or feeling just by looking at their face*
- I find it difficult to work out the intentions of other people

*Reverse scored
Exclude: any sum score of 4 or more.

Have you flown abroad in the last month? (alternatives: yes, no)
Follow-up: yes [exclude if it was less than three weeks since this trip or the location was less than three time-zones away]

Johns, M. W. A new method for measuring daytime sleepiness: the Epworth sleepiness scale. Sleep, 1991, 14: 540–545
Chung, F., Abdullah, H.R., Liao, P. STOP-bang questionnaire a practical approach to screen for obstructive sleep apnea. Chest, 2016, 149: 631–638.

Supplementary tables

Table S1. *Bayesian regression estimating model-building performance by sleep deprivation (2 pairs removed)*

	Describer sleep deprivation effect				Builder sleep deprivation effect			
Outcome	Mu	HDI low	HDI high	Sigma	Mu	HDI low	HDI high	Sigma
Score	**0.86**	**0.07**	**1.69**	**0.41**	**-1.10**	**-1.91**	**-0.32**	**0.41**
Time taken (seconds)	-7.06	-49.31	35.22	21.91	14.47	-27.77	57.39	21.71
Efficiency	0.31	-0.06	0.73	0.20	-0.40	-0.80	0.02	0.21

Note. Sleep-deprived describer N = 44; Control describer N = 45; Sleep-deprived builder N = 43; Control builder N = 46. Mu = posterior distribution mean; Sigma = posterior distribution residual standard deviation; HDI = 95% Highest Density Interval; Bold rows represent changes in performance that can be considered 'significant'. Score model priors: mean effect = 0, sigma = 2.25, limited between -9 and 9. Time-taken priors: mean effect = 0, sigma = 150, limited between -600 and 600. Efficiency model priors: mean effect = 0, sigma =0.5, limited between -2 and 2. A cumulative distribution was used for the score response outcome, while a Gaussian distribution was used for time and efficiency outcomes.

Table S2. *Bayesian multilevel regression estimating word-description performance by sleep deprivation (2 pairs removed)*

	Speaker sleep deprivation effect				Guesser sleep deprivation effect			
Outcome	Mu	HDI low	HDI high	Sigma	Mu	HDI low	HDI high	Sigma
Score (per round)	0.06	-0.37	0.47	0.21	-0.10	-0.51	0.31	0.21

Note. Sleep deprivation N = 89; Control N = 91. Mu = posterior distribution mean; HDI = 95% Highest Density Interval; Sigma = posterior distribution residual standard deviation. Priors were set on the effect; since there was no maximum possible effect, sigma was set as a quarter of maximum observed round score (mean = 0, sigma = 1.5). The response distribution was set to cumulative since model fit comparisons revealed that this was the best fit for the data.
